# Superparamagnetic Fe_3_O_4_-PEG_2K_-FA@Ce6 Nanoprobes for *in Vivo* Dual-mode Imaging and Targeted Photodynamic Therapy

**DOI:** 10.1038/srep36187

**Published:** 2016-11-08

**Authors:** Ting Yin, Peng Huang, Guo Gao, Joseph G. Shapter, Yulan Shen, Rongjin Sun, Caixia Yue, Chunlei Zhang, Yanlei Liu, Sui Zhou, Daxiang Cui

**Affiliations:** 1 Institute of Nano Biomedicine and Engineering, Shanghai Engineering Research Center for Intelligent Diagnosis and Treatment Instrument, Department of Instrument Science and Engineering, Department of Micro/Nano Electronics, School of Electronic Information and Electrical Engineering, Shanghai Jiao Tong University, 800 Dongchuan Road, Shanghai 200240, P.R. China; 2 School of Chemical and Physical Sciences, Flinders University, Bedford Park, Adelaide 5042, Australia; 3 Department of Radiology, Shanghai Jiao Tong University Affiliated Sixth People’s Hospital, Shanghai Jiao Tong University, Shanghai, 200240, China

## Abstract

The development of targeted nanoprobes is a promising approach to cancer diagnostics and therapy. In the present work, a novel multifunctional photo/magnet-diagnostic nanoprobe (MNPs-PEG_2K_-FA@Ce6) has been developed. This nanoprobe is built using folic acid (FA), bifunctional polyethylene glycol (PEG_2K_) and photosensitizer chlorin e6 (Ce6). The MNPs-PEG_2K_-FA@Ce6 nanoprobes are superparamagnetic, can be synthesized on a large scale by a one-pot hydrothermal process without further surface modification and are stable in an aqueous environment for eight months. Compared with free Ce6 nanoprobes *in vitro* studies, the MNPs-PEG_2K_-FA@Ce6 nanoprobes significantly enhance cellular uptake efficiency and promote the effectiveness of photodynamic therapy (PDT) with the assistance of 633 nm laser irradiation. The unique nanoprobes show superior penetration and a retention time of more than six days with less accumulation in the liver allowing highly effective tumor recognition and monitoring. Additionally, there was little damage to healthy organs or tissues. These exciting new nanoprobes could be potential building blocks to develop new clinical therapies and translational medicine.

In the last few years, gastric tumors have emerged as the secondary cause of cancer-associated mortality globally^1^,^2^,^3^, and there has been a significant increase compared to other malignant tumors in China according to the latest cancer statistics^4^. Effective tools for early detection of and therapy for cancer, especially in high-risk individuals, are desperately required for minimizing the morbidity and mortality rates^5^,^6^.

Photodynamic therapy (PDT) that employs the singlet oxygen generated by photosensitizer (PS) molecules under light exposure to cause irreversible damage to malignant cells is newly used to cure carcinoma in the clinic^7^. PDT has generated great interest because of its essential merits such as selective, noninvasive and repeatable treatment. Unfortunately, there are several significant obstacles for free photosensitizers including water-insolubility and poor pharmacokinetics, which have greatly limited the clinical application of PDT therapy. Recently, the application of advanced nanotechnology for diagnostics and therapy has attracted great attention^8^. The majority of nanoparticles (NPs) modified by numerous organic and inorganic matters with long circulation times have been used as photosensitizer delivery carriers because such agents could efficiently accumulate in tumor tissues due to their enhanced permeability and retention (EPR) effects^1^,^9^,^10^. Based on EPR effects, a passive targeting strategy of tumors leads to extensive pathophysiological heterogeneity^1^. Molecular targeting is a type of well-established personalized medical method, which relies on exclusively identifying and binding between active targeting ligands anchored on the nanoparticle surface and over-expressed folate receptors on the membrane of tumor vasculature or tumor cells. FA-mediated Fe_3_O_4_ NPs can not only be specifically uptaken by cancer cells, but also be used for efficient targeted magnetofluorescent imaging of a gastric cancer mouse model. Dual modal imaging refers to the incorporation of two medical imaging modalities using the same probe, which could provide better insights into physiological mechanisms at molecular and cellular levels^11^,^12^. Recently, a combination of magnetic resonance imaging (MRI) and optical imaging platforms for cancer diagnostics and therapy have attracted great interest^13^,^14^. The noninvasive MRI imaging could provide multidimensional structural, functional and morphological information for human/animal soft tissue imaging contrast^15^, and great advances have been achieved for MRI based molecular optical imaging and cell-labeled tracking^16^,^17^. Similarly, unparalleled structural detail can be achieved by fluorescent imaging, which is attributed to photosensitizer molecules. It is useful to combine MRI and fluorescent imaging into a synergistic imaging tool for precisely visualizing and demarcating structural/functional details before PDT treatment^11^,^18^. Based on targeted NPs and integration of diagnosis and treatment system, there will be more useful and viable approaches to fight cancer^19^,^20^.

In our previous work, we applied some multifunctional diagnosis platforms using single photosensitizer-conjugated/loaded magnetic nanoparticles^14^ or gold nanoclusters^21^ for fluorescence imaging to monitor in real time and guide photodynamic therapy (PDT). Targeting and tracking gastric cancer cells *in vivo* is realized by labelling fluorescent magnetic nanoparticles (MNPs) with marrow mesenchymal stem cells^22^. Although some progress has been made, there are still many challenging problems including the nanoprobes’ safety and targeted tumor efficiency while not leading to significant accumulation in the liver or spleen. Therefore, minimizing the accumulation amounts of nanoprobes in the liver or spleen while ensuring effective targeted imaging is a great interest for clinical application of early diagnosis of gastric cancer.

Herein, we have developed a facile one-pot hydrothermal route for large-scale synthesis of highly stable superparamagnetic Fe_3_O_4_ nanoparticles (7.15 ± 1.3 nm in diameter) containing carboxyl groups which do not require further surface modification. Next, the Fe_3_O_4_ nanoparticles, polyethylene glycol (PEG_2K_)-coated, and folic acid (FA)-functionalized nanoprobes were incorporated with the photosensitizer of chlorin e6 (Ce6) to ultimately yield the MNPs-PEG_2K_-FA@Ce6 nanoprobes. The schematic is shown in Fig. 1. The introduction of flexible PEG_2K_ can enhance cell surface recognition, reduce the nanoparticles agglomeration and protein adsorption, extend circulation time and improve the efficiency of targeting and internalization *in vivo*^23^. Human gastric carcinoma MGC-803 cells and normal gastric epithelial cells (GES-1) were used in the experiments. MRI and fluorescent dual mode imaging were carried out in order to evaluate the synthesized MNPs-PEG_2K_-FA@Ce6 targeted nanoprobes and explore the potential for future clinical applications. Our results demonstrated that the synthesized MNPs-PEG_2K_-FA@Ce6 nanoprobes are effective for *in vivo* targeting, lead to less accumulation in the liver while having superior penetration and longer retention time in naive female nude mice.

## Material and Methods

### Materials

Ferric citrate, FeSO_4_·7H_2_O, Folic acid (FA), acetone and tetrahydrofuran (THF) were supplied by Sinopharm Chemical Reagent Co., Ltd. (Shanghai, China). Ascorbic acid (AA), N-hydroxysuccinimide (NHS), chlorin e6 (Ce6) and 1-(3-Dimethylaminopropyl)-3-ethylcarbodiimide hydrochloride (EDC) were from Aladdin Chemical Reagent Co., Ltd. (Shanghai, China). Amine-PEG_2K_-Amine (H_2_N-PEG_2K_-NH_2_, MW≈2K) was obtained from Shanghai XiBao Biotechnology Co., Ltd. (Shanghai, China). 3-[4,5-dimethylthiazol-2-yl]-2,5-diphenyltetrazolium bromide (MTT) and Hoechst 33342 were from Sigma-Aldrich Chemical Co., Ltd. (St Louis, MO). MGC-803 cells and GES-1 cells were kindly provided by Chinese Academy of Science. Unless otherwise stated, all cell culture medium were purchased from GIBCO (Shanghai, China). The water used in all experiments was ultrapure water, which was purified using a Milli-Q Water System (18.2 MΩ cm, Millipore Co., USA).

### Synthesis of MNPs-COOH

Typically, a FeSO_4_·7H_2_O solution was mixed with ferric citrate solution in the molar ratio of 2:1. After intense sonication for 10 min, 0.1 g ascorbic acid (AA) was added to the mixture, and the pH was adjusted to 10 using 0.4 M NaOH solution after which the mixture was vigorously stirred for 20 min with a magnetic apparatus. After that, the black/green precursors were transferred into a 50 mL capacity Teflon-lined autoclave, sealed tightly and raised to 200 °C at a rate of 1.5 °C/min, then kept at 200 °C for 10 h. After the autoclave cooled to room temperature, the products were purified using Amico Ultra Centrifugal Filters (MWCO 10 kDa; Millipore) in a swing bucket rotor at 11300 rpm for five minutes, washed several times to remove salt and/or other by-products. Finally, the black products were dissolved using 50 mL ultrapure water, and then kept at 4 °C in refrigerator for later use.

### Synthesis of activated FA

NHS-activated FA (FA-NHS) was prepared according to a reported procedure^24^. In brief, 1.5 g FA was dissolved in a mixture of TEA (0.6 mL) and DMSO (50 mL) at 45 °C with mild stirring. To the resulting solution, 0.52 g DCC and 0.55 g NHS were added orderly, and the mixture was stirred for 20 h in the dark at room temperature. The products were purified via filtration of reaction mixture, and insoluble dicyclohexylurea was removed through a glass wool plug. The filtrate was washed four times with diethyl ether/acetone and then the precipitates were collected via centrifugation. The freeze-dried pellet was stored at −20 °C in the freezer for later use.

### Synthesis of MNPs-PEG_2K_-FA@Ce6

MNPs were reacted with NH_2_-PEG_2K_-NH_2_ and FA by an EDC/NHS coupling reaction to form the MNPs-PEG_2K_-NH_2_ and MNPs-PEG_2K_-FA. A typical reaction of NHS-activated MNPs (MNPs-NHS) was as follows: EDC (16 mg) and NHS (20 mg) were sequentially added to 2 mL MNPs in MES solution (keeping the solution at pH 5.0) and the reaction components were mixed well and left to react at room temperature for 30 min to activate the carboxyl group of MNPs. The MNPs-NHS was washed four times using tetrahydrofuran by repeatedly resuspending the suspension and then collecting the products using high speed centrifugation. The collected pellet was re-dissolved with 2 mL borate saline buffer solution (keeping the solution at pH 7.2). MNPs-PEG_2K_-NH_2_ was prepared. Following the previous steps, 12 mg PEG_2K_ (Amine-PEG_2K_-Amine, MW≈2000) was quickly added to 2 mL of MNPs-NHS solution by vigorously stirring for 12 h at room temperature. After the end of reaction, the products were dissolved using a similar purification procedure as the previous method. MNPs-PEG_2K_-FA was prepared in BS solution. Under the same reaction conditions, 4 mg FA-NHS was dissolved in 2 mL of MNPs-PEG_2K_ solution followed by constant stirring for 20 h. It was purified using an Amicon Ultra Centrifugal Filters with an MWCO of 10 kDa and washed four times with PBS solution (pH = 7.4) to remove unconjugated folic acid. In order to incorporate the Ce6 molecules, 2 mg Ce6 dissolved in 1 mL of 0.1 M NaHCO_3_ was mixed with 8 mg MNPs-PEG_2K_-FA with 5 mL of 0.1 M NaHCO_3_ in a shaking bed at room temperature overnight. Thereafter, the reaction products were concentrated with an MWCO 10 kDa of Amico Ultra Centrifugal Filters. The filtrates were collected to calculate the loading amount of Ce6 molecules. The collected pellet was re-dissolved in 500 μL of PBS solution.

### Characterization of MNPs and MNPs-PEG_2K_-FA@Ce6

The morphologies of the MNPs and hybrid nanoprobes were observed using TEM with a JEM-2100F (JEOL, Japan). The crystalline compositions of the as-prepared MNPs were characterized by an X-ray diffractometer with Cu Kα radiation (λ = 0.15418 nm) at a 2θ scan range of 20–70° and a scanning rate of 10 degrees per min (D8 ADVANCE, BRUKER-AXS). Thermal gravimetric analysis (TGA/DSC) was measured using a Pyris 1TGA (Perkin Elmer, America) in N_2_ atmosphere at a heating rate of 20 °C/min. The number of carboxyl groups on the MNPs were measured via digital conductivity instrument (ULTRAMETER II TM 4P, Myron L Company, USA). UV–vis spectra of MNPs, MNPs–PEG_2K_, MNPs-PEG_2K_-FA, free Ce6 and MNPs-PEG_2K_-FA@Ce6 were recorded on a Varian Cary 50 spectrophotometer (Varian Inc., Palo Alto, CA, USA). Fluorescence spectra (PL) of MNPs, free Ce6 and MNPs-PEG_2K_-FA@ Ce6 samples were measured with a Hitachi FL-4600 spectrofluorometer. Fourier transform infrared (FTIR) spectra were performed using a Nicolet 6700 FTIR spectrometer using KBr-supported pellets (Thermo Electron Corporation, Madison, WI, USA). Magnetization loops were done by a Physical Property Measurement System (PPMS) EverCool-II with 9 Tesla magnets (Quantum Design, USA). The T2 relaxation times of progressive concentration of the two samples were obtained by using a 1.4 T Bruker Minispec Analyzer (MQ60). In brief, the various concentration of contrast agent (200 μL, ultrapure water medium) were shifted in dedicated sample tubes, and immersed in a 37 °C circulating water bath pot to remain constant. Subsequently, the r2 value (^mM-1 s-1^) of MNPs and MNPs-PEG_2K_-FA@Ce6 NPs was received by the fitting of T2 value corresponded to the serial concentration (mM) plots, the slope of the optimum fitting straight, namely.

### Drug loading and release efficiency measurements

The theoretical drug loading percentage was calculated according to the equation 1:


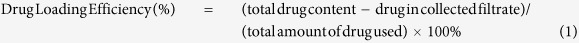


The release of Ce6 from MNPs-PEG_2K_-FA@Ce6 was measured with UV-Vis spectroscopy placed in shaking reactor at 37 °C in PBS buffer (pH = 7.4), using a slightly modified procedure from that previously reported^22^. In brief, the 2 mL of MNPs-PEG_2K_-FA@Ce6 dispersion (16 mg mL^−1^) was put into a dialysis tube (MWCO 3500) and dipped in 50 mL of the PBS media with constant shaking (400 rpm) at 37 °C. At pre-set times, 2 mL release solution was transferred for UV–vis detection and compensated with similar volume of fresh solution.

### Cell Culture and Cytotoxicity Assay

The MGC-803 and GES-1 cells were regularly cultured in 25 cm^2^ plates at 37 °C in a humidified incubator with 5% CO_2_, immersed in 8 mL of Dulbecco’s Modified Eagle’s Medium (DMEM) containing 10% (vol/vol) heat-inactivated fetal bovine serum (FBS), penicillin (100 U mL^−1^) and streptomycin (100 μg mL^−1^). Then, the photo-therapeutic efficacy *in vitro* of hybrid nanoprobe was checked by an MTT viability assay of MGC-803 cells. Briefly, 5 × 10^3^ cells were seeded in 96-well plate to adhere the cells. After a 12 h incubation, the medium was discarded instead of 100 μL fresh medium such as PBS (control group), cell culture fluid containing different concentrations of MNPs (with final concentration of 5–100 μg mL^−1^) and MNPs-PEG_2K_-FA@Ce6 was added. Subsequently, the cells were washed twice using fresh medium after co-incubation 12 h. Then, each hole in 96-well plate was irradiated using or not using 633 nm helium-neon (He-Ne) laser at a maximum power of 50 mW/cm^2^ for 1 min to examine phototoxicity and dark toxicity of free Ce6 and MNPs-PEG_2K_-FA@Ce6. After 24 h incubation under common conditions, MTT solution was put into each well and the MGC-803 cells and GES-1 cells were cultured for additional 4 h. After that, 100 μL of DMSO was used to dissolve the solid formazan crystals. The optical density (OD) value of per well was tested using a Multiskan mircoplate reader (Thermo Scientific, Thermo MK3) at 570 nm. The average and standard deviation (SD) of six parallel wells for each concentration were reported.

### Cellular uptake *in vitro*

To observe the specific uptake of the nanoprobe, both MGC-803 and GES-1 cells were respectively seeded into 4-chamber glass bottom dish to adhere the cells. After overnight incubation, using the free Ce6 (10 μg mL^−1^) and MNPs-PEG_2K_-FA@Ce6 (equivalent Ce6 10 μg mL^−1^) solution repeatedly, the adherent cells were adequately rinsed using PBS and fixed with paraformaldehyde (4%) for 20 min at 4 °C in a refrigerator. The nuclei of the cells were counterstained according to a standard procedure of Hochest (1 mg mL^−1^) for 20 min. The fluorescence signal of free Ce6 and the fluorescent nanoprobes in cells were also examined. The emission was separately collected through either 420–460 nm or 650–700 nm barrier filter. The targeting specificity of the nanoprobes to MGC-803 cells was recorded using flow cytometry by monitoring the fluorescence signal in FL3-H (λ_em_ = 650–700 nm) channel. Both MGC-803 and GES-1cells were cultured overnight in 12-well plates at a density of 5.0 × 10^4^ cells per well. Then the cells were coincubated using free Ce6 or MNPs-PEG_2K_-FA@Ce6 for various times. Finally, the adherent cells were rinsed twice using PBS, and then trypsinized, centrifuged, re-suspended in PBS solution and recorded by flow cytometer.

### Animal model loaded with gastric cancer

Animal procedures were carried out in accordance with the relevant guidelines and regulations approved by institutional animal use, care and ethics committee. Female nude mice (20–22 g weight) were obtained from SLAC Laboratory Animal center (Shanghai, China). Animals were raised five in each cage at 22 ± 2 °C, fed a normal diet and water, and kept in animal care protocols under pathogen-free conditions. The life sciences committees of Shanghai Jiao Tong University approved the experiments, and all animal procedures were in agreement with institutional animal use and care regulations. About 4 weeks later, an animal tumor model (10–15 mm in diameter) was established by subcutaneously injecting 5 × 10^6^ MGC-803 cells resuspended in PBS solution (0.1 mL) on the right hind leg area of each nude mice (6 weeks old).

### *In vitro*/vivo fluorescent imaging and MR imaging

After intravenous injection of MNPs-PEG_2K_-FA@Ce6 (10 μg mL^−1^ Ce6) by the tail vein, the real-time *in vivo* optical imaging was carried out by the Signa HDx (GE medical systems, U.S.). All *in vitro*/*vivo* MR scanning were carried out with a 3.0 T Siemens Magnetom Trio medical MR systems (Shanghai sixth people’s hospital radiology department of MRI, Shanghai, China) with the following parameters: TR = 2600 ms, slice thickness = 2 mm, TE = 52 ms, matrix = 320 × 256, FOV = 1 cm × 1 cm, NEX = 2, and acquisition time 96 s. The gray values of both MGC-803 and GES-1 cells at varying Fe concentrations (0.16, 0.32, 0.64, 1.28 and 2.56 mM, respectively) were performed using a clinical MR system and obtained with MR imaging software. The nude mice were anesthetized, placed inside a receiver coil and scanned after post-injection 12 h and 15 h of MNPs or MNPs-PEG_2K_-FA@Ce6.

### Photodynamic therapy of gastric cancer *in vivo*

Right-hand MGC-803 subcutaneous xenografted tumor models were used to estimate the effect of photodynamic therapy *in vivo*. Firstly, the experiment mice were randomly divided into five groups of five mice/group, when the size of tumor reached ~100 mm^3^. Briefly, (1) PBS (100 μL) with laser (control group), (2) free Ce6 (5 mg/kg), (3) free Ce6 (5 mg/kg) combined with laser irradiation, (4) MNPs-PEG-FA@Ce6 (doses equivalent to 5 mg/kg of Ce6), (5) MNPs-PEG_2K_-FA@Ce6 (doses equivalent to 5 mg/kg of Ce6) upon laser irradiation were respectively injected by caudal vein. In exposure groups, a 633 nm He-Ne laser was used with 50 mW/cm^2^ for 30 min after 10 and 24 h post-injection. Then, the tumor size and body weight of each rat were measured every 3 days after treatment until 18 days. At day 18, rats treated with MNPs-PEG_2K_-FA@Ce6/laser were sacrificed. The major organs and tissues (liver, spleen, lungs, kidneys and heart) collected from gastric tumor-bearing mice at day 18 after injection in MNPs-PEG_2K_-FA@Ce6/laser treatment group were sliced, stained with hematoxylin and eosin (H&E) as routine, then morphological characteristics of each organ tissue were observed with microscopy.

## Results and Discussion

### Synthesis and characterization of MNPs

**Figure 2:**
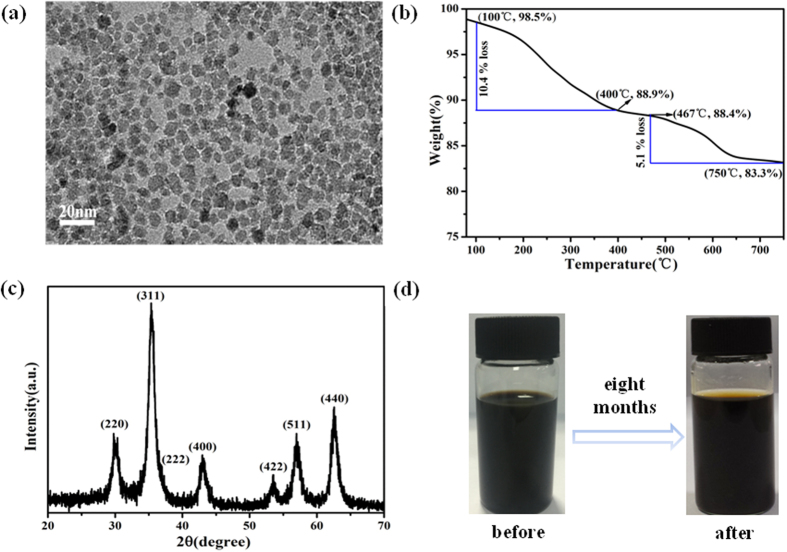
Characterization of the as-formed MNPs (**a**) TEM image, (**b**) TGA analysis, (**c**) XRD pattern, (**d**) visual image of Fe_3_O_4_ nanoparticles dispersed in ultrapure water.

The TEM image (Fig. 2a) shows that the Fe_3_O_4_ nanoparticles were uniform in size and have a spherical structure with a diameter of about 7.15 ± 1.3 nm (Figure SI1). The thermal stability and decomposition behavior of MNPs were evaluated by thermo-gravimetric analysis (TGA) in a nitrogen atmosphere, as shown in Fig. 2b. The weight loss from 100 to 400 °C corresponds to the removal of the unstable oxygen-containing groups from the organic species. The further weight loss in the range of 467 to 750 °C was related to the removal of the relatively stable oxygen-containing groups. When the temperature is over 750 °C, the oxygen-containing groups on the surface of Fe_3_O_4_ are completely removed. The total weight loss of 16.7% reveals that the surface of the synthesized Fe_3_O_4_ nanoparticles has large numbers of carboxyl groups. The amount of carboxyl groups on the surface of Fe_3_O_4_ nanoparticles, obtained from the conductometric titration curve (Figure SI2), is 0.73 mmol/g (more details in Supporting information)^25^,^26^. The synthesized brownish black products were dried and characterized through X-ray power diffraction (XRD). The XRD pattern (Fig. 2c) of the samples is consistent with that of the standard cubic Fe_3_O_4_ structure (JCPDS no.19-0629). Based on the strongest peak (311), the size of nanoparticles is 7.85 nm according to the Scherrer formula, which is in agreement with the TEM data. It is interesting to observe that the synthesized MNPs exhibited excellent water dispersibility. Eight months after the initial synthesis, there were no precipitates at the bottom of the bottle (Fig. 2d).

**Figure 3:**
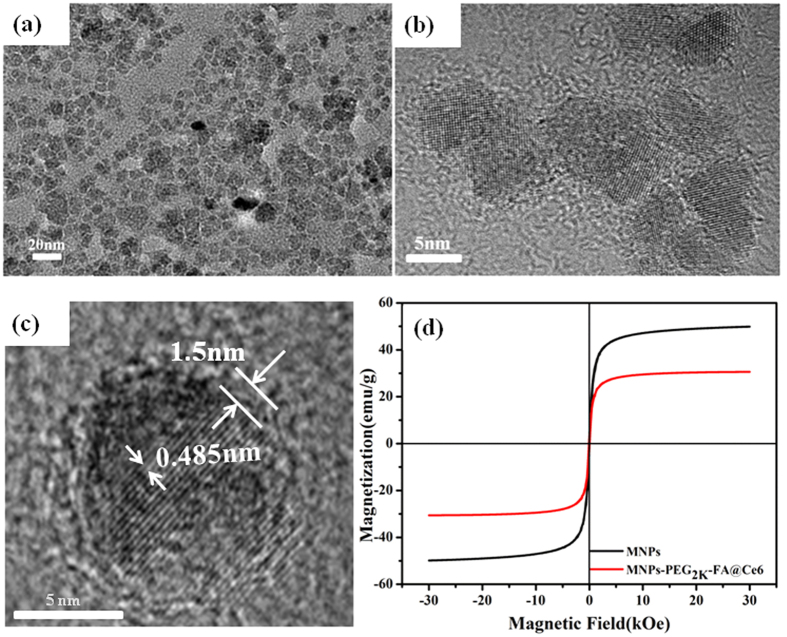
Characterization of the MNPs-PEG_2K_-FA@Ce6 nanoprobes. (**a**) TEM image, (**b**,**c**) HRTEM pattern, (**d**) magnetization loops measured at 300 K.

The hydrodynamic sizes of MNPs, MNPs-PEG_2K_, MNPs-PEG_2K_-FA, MNPs-PEG_2K_-FA@Ce6 dissolved in ultrapure water were measured to be 39.3 nm, 54.7 nm, 75.5 nm and 72 nm respectively as determined by dynamic light scattering (DLS). In addition, the Zeta-potential of MNPs, MNPs-PEG_2K_, MNPs-PEG_2K_-FA, MNPs-PEG_2K_-FA@Ce6 dispersed in ultrapure water were measured to be −3.59 mV, 3.46 mV, −0.95 mV and −3.37 mV. To estimate the stability of the MNPs and MNPs-PEG_2K_-FA@Ce6 nanoprobes, they were dispersed in PBS with a pH of 7.4. It was shown that the systems were stable and that there was no aggregation after 1 d (Figure SI3). The hydrodynamic sizes of MNPs and MNPs-PEG_2K_-FA@Ce6 in PBS were measured to be 30.3 nm and 159.8 nm, which was significantly smaller than the iron oxide nanoprobes reported previously^27^. The TEM image of Fig. 3a shows the MNPs-PEG_2K_-FA@Ce6 nanoprobes are highly dispersed. Figure 3b shows the distinct and successive lattice-fringe images, indicating the perfect single-crystal of Fe_3_O_4_. The fringe space between adjacent fringes is measured to be 0.485 nm, which is close to the (111) crystalline plane of Fe_3_O_4_. In order to improve the biocompatibility and loading of the fluorescent dye (Ce6), NH_2_-PEG_2K_-NH_2_ was used to modify the nanoparticle surface. The thickness of coating layer was about 1.5 nm (Fig. 3c). Figure 3d showed that MNPs-PEG_2K_-FA@Ce6 nanoprobes exhibit superparamagnetic properties. The saturation magnetization value (Ms) of the nanoprobes is observed to be 30.6 emu g^−1^. These results show that the prepared MNPs-PEG_2K_-FA@Ce6 are small and can be applied in MRI for clinical diagnosis^28^.

### Formation and characterization of MNPs-PEG _2K_-FA @Ce6 nanoprobes

**Figure 4:**
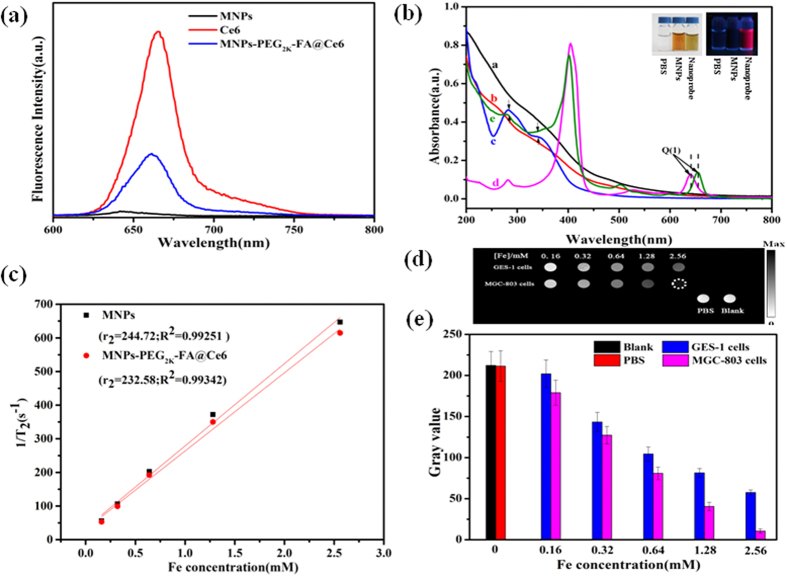
Characterization of the MNPs-PEG_2K_-FA@Ce6 nanoprobes. (**a**) PL spectra, (**b**) UV−Vis absorption. (a. MNPs, b. MNPs-PEG_2K_, c. MNPs-PEG_2K_-FA, d. Ce6, and e. MNPs-PEG_2K_-FA @Ce6), (Insets) Digital pictures of PBS, MNPs, free Ce6, and MNPs-PEG_2K_-FA@Ce6 dispersed in PBS solution (the left of photograph was under white-light, and the right of one was under UV (365 nm) excitation), (**c**) the value of T2 relaxation rate of MNPs and MNPs-PEG_2K_-FA@Ce6 NPs under various Fe concentration, (**d**) the T2-weighted MR images of MNPs-PEG_2K_-FA@Ce6 nanoprobes under various Fe concentration and (**e**) the gray value in GES-1 and MGC-803 cells.

On the basis of the stability of as-synthesized MNPs dispersed in aqueous solution, the highly stable intermediate products of MNPs-PEG_2K_-FA were formed, and then the drug (Ce6) was embedded within PEG_2K_ network. The Ce6 molecules were stable in the intermediate products due to hydrophobic interactions between nonpolar segment of Ce6 and PEG_2K_ chains^21^. The loading capacity of Ce6 molecules in the intermediate products was about 10.16 wt%, based on UV-Vis spectroscopy analysis. Owing to the resonance energy transfer, the fluorescence of nanoprobes was partially quenched (Fig. 4a). Figure 4b is the absorption spectra of MNPs, MNPs-PEG_2K_, MNPs-PEG_2K_-FA, free Ce6 and MNPs-PEG_2K_-FA@Ce6 in PBS. The Curve b in Fig. 4b shows the characteristic peak which has a slightly blue-shift to 276 nm after PEG_2K_ was conjugated to the MNPs, compared with PEG_2K_ (characteristic absorption peak around 283 nm). It may be attributed to the change of polarization rate of molecule? Particularly, there are two wide distinct peaks at 350 nm and a sharp peak at 283 nm, which are the characteristic absorption peaks of FA in the MNPs-PEG_2K_-FA and MNPs-PEG_2K_-FA@Ce6^29^. Importantly, it was shown that there is a pronounced red shift in the absorption from 645 nm (curve d in Fig. 4b) to 654 nm (curve e in Fig. 4b) in the region of Ce6 Q(I) band, which was a typical response of Ce6 in different chemical environment^21^. Interestingly, the spectra of Ce6 (sample d) in Fig. 4b also had a little sharp peak around 283 nm. It may be caused by containing the carbonyl group as chromophore, which was possibly attributed to n electronic transition of impurity atoms on unsaturated bond into П* orbit^30^. However, it belongs to the weak absorption band, as shown a little sharp peak. FTIR spectral measurements showed that the components of the nanoprobes such as FA, PEG_2K_ and Ce6 were combined with the MNPs as expected (Figure SI4). The FTIR shows that the PEG_2K_ is bound to the MNPs by the formation of an amide with the vibrations for the amide bond observed at 1647 cm^−1^ (C=O) and 1533 cm^−1^ (N–H). The peak at 1108 cm^−1^ (C–O–C), 2851 and 2919 cm^−1^ (–CH_2_–) indicated the existence of PEG_2K_. Additionally, the peak at 1604 cm^−1^ is the characteristic absorption peak of amino group from pteridine ring of FA, suggesting the successful chemical coupling of MNPs-PEG_2K_ and FA. The small particle size results in the superparamagnetic behaviour^31^. The membrane surface of GES-1 cells without FA over-expression receptor were as control group. After incubation for 12 h with the MNPs-PEG_2K_-FA@Ce6 nanoprobes at different Fe concentrations, the MGC-803 and GES-1 cells were subjected in 1 × PBS to T2-weighted MR imaging at room temperature (Fig. 4d,e). It was shown that the gray value of both GES-1 and MGC-803 cells reduced with increasing Fe concentration. Apparently, the reducing trend of MGC-803 cells was much more than that of GES-1 cells under the same experimental conditions (Fig. 4d). To further prove T2 signal enhancement effect following the increase of the Fe concentration (Fig. 4c), quantitative statistics of the gray value are shown in Fig. 4e, suggesting the feasibility of the synthesized hybrid nanoprobes for medical diagnosis.

### Cellular Uptake Assay

**Figure 5:**
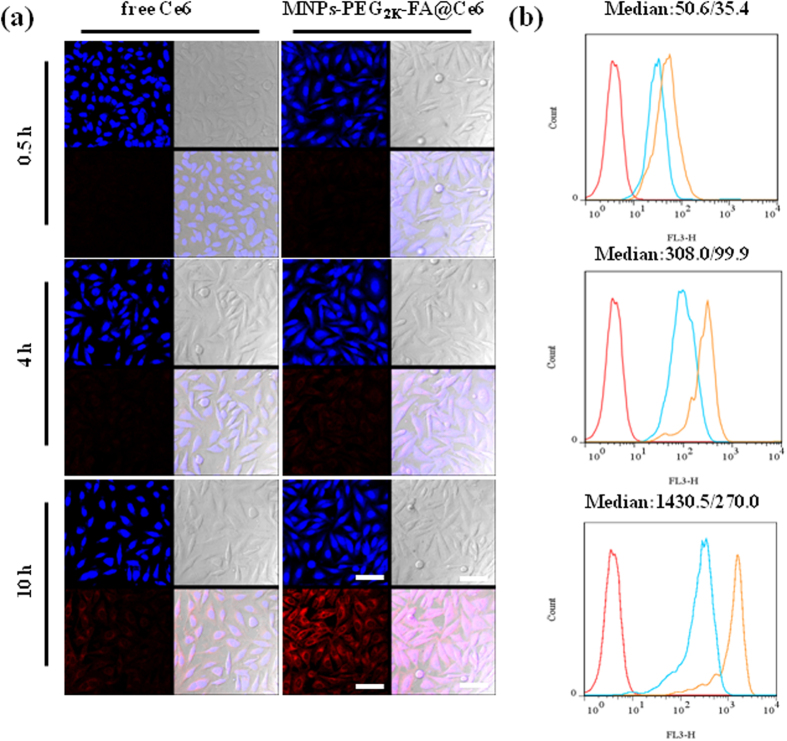
(**a**) Confocal images showing relationship between MGC-803 cells and free Ce6 and MNPs-PEG_2K_-FA@ Ce6 for 0.5, 4, and 10 h. (Note that: Each block diagram is composed of four small squares. The blue areas in top left of four small squares shows the nucleus of fluorescent images; lower left image was fluorescent images about cellular uptake of MNPs-PEG_2K_-FA@Ce6 or free Ce6 in red field; top right images are of MGC-803 cells in light field; lower right are merge images of the other three small squares). The scale bar was 100 μm. (**b**) Flow cytometry graphs showing significant variation of cellular uptake between targeted nanoprobes and no-targeted nanoprobes. Red, cyan and orange curves represent interactions of PBS, free Ce6 and MNPs-PEG_2K_-FA@Ce6 with MGC-803 cells, respectively. (In median: X/Y, X was representative for the amounts of cellular uptake of control group, and Y was representative for the amounts of cellular uptake of MNPs-PEG_2K_-FA@Ce6 nanoprobes).

Various samples with and without FA ligands were used to interact with gastric cancer MGC-803 cells and GES-1 cells. Both the confocal laser scanning microscopy (CLSM) and flow cytometry (FCM) were carried out to evaluate their targeted effectiveness in Fig. 5. The fluorescence intensity of the MGC-803 cells incubated with MNPs-PEG_2K_-FA@Ce6 (equivalent Ce6) and free Ce6 was monitored for 10 h. With increasing incubation time, the MNPs-PEG_2K_-FA@Ce6 were taken up much more dramatically than free Ce6 in the MGC-803 cells (Fig. 5a). After a half an hour incubation, the fluorescence signals of the cells that had interacted with the MNPs-PEG_2K_-FA@Ce6 were brighter than with free Ce6, suggesting much more Ce6 has been endocytosed into the cells. Furthermore, the fluorescent distribution was very uniform in the cytoplasm and cytomembrane. This result was also confirmed by the quantitative analysis of fluorescence intensity by FCM. The median values of the fluorescence intensity for the cells incubated with the nanoprobes at 0.5 h was about 1.4-fold, compared with control group (free Ce6) in Fig. 5b. After 10 h of successive incubation, the different quantity of phagocytosis between MNPs-PEG_2K_-FA@Ce6 and free Ce6 was most significant, and then the median values of the fluorescence intensity was about 4.3-fold higher. To further evaluate the specific targeting of the nanoprobes, we chose FR-negative cells such as GES-1 cells to assess the interaction of the cells and the nanoprobes as another control group in Figure SI5. It was shown that the fluorescence intensity of GES-1 cells incubated with free Ce6 increased remarkably from 4 h to 10 h. However, the weaker red fluorescence signal was displayed even after 10 h the GES-1 cells incubated with the nanoprobes, compared with interaction effect for MGC-803. These results were due to over-expression of membrane folate receptors such as MGC-803 cells.

### *In Vitro* Cellular Toxicity

**Figure 6:**
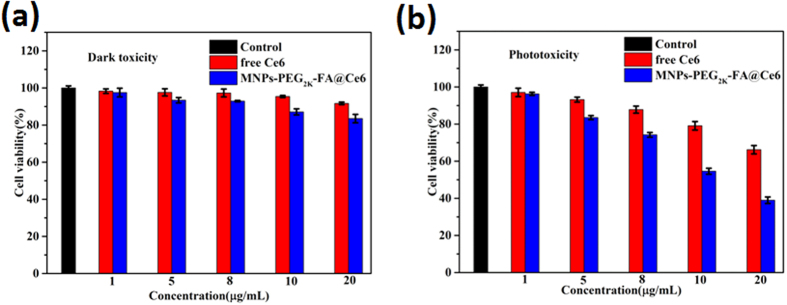
The toxicity analysis in the dark (**a**) and under the laser irradiation (λ_ex_ = 633 nm) (**b**) of free Ce6 and MNPs-PEG2K -FA @ Ce6 incubation with MGC-803 cells.

To evaluate the photocytotoxicity of free Ce6 and the nanoprobes under the laser irradiation (λ_ex_ = 633 nm), MTT assay and fluorescence microscopy were carried out respectively. Figure 6 and Figure SI8 indicated that the MNPs-PEG_2K_-FA@Ce6 nanoprobes had the best biocompatibility and non-toxicity toward the MGC-803 cells and GES-1 cells in the absence of laser irradiation by MTT assay. However, it was worth noting that the MGC-803 cell death ratios caused by the nanoprobes (10 μg/ml of Ce6 equiv) under the laser irradiation were about 1.5-fold higher than control group (10 μg/ml of free Ce6), while GES-1 cell death ratios were about 1.3-fold higher than control group. The prominent photocytotoxicity of the MNPs-PEG_2K_-FA@Ce6 could be attributed to the increase of cellular uptake, and subsequently generate much more reactive oxygen species (ROS) (Figure SI6). Figure SI7 demonstrates the non-toxicity of MNPs-based nanoprobes without the laser irradiation. The amount of drug released is shown in Figures SI9 and SI10 reaching a release of about 30% after 6 h. The hydrophobic Ce6 molecule loaded into nanoprobes showed excellent stability and biocompatibility, indicating great potential as feasible nanoprobes for PDT treatment *in vivo*. These results were consistent with the detection of photodamage by fluorescence microscopy (Figure SI11).

### *In vivo* fluorescent imaging and MR imaging

**Figure 7:**
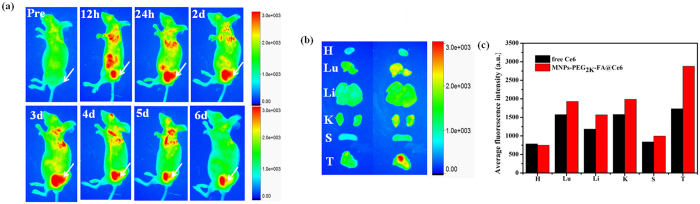
(**a**) Representative fluorescence images of tumor-bearing mouse after injection with MNPs-PEG_2K_-FA@Ce6 by real-time observation for 6 d. (**b**) *Ex vivo* fluorescence imaging of major organs and tumors of mice dissected at 24 h post-injection of free Ce6 and MNPs-PEG_2K_-FA@Ce6. The letters H, Lu, Li, K, S and T stand for heart, lung, liver, kidney, spleen and tumor respectively. (**c**) The quantitative fluorescent analysis of the dissected organs and tumors of mice after tail vein injection of 24 h.

To evaluate tumor specific targeting property of MNPs-PEG_2K_-FA@Ce6 nanoprobes, the whole-body optical imaging system was applied to monitor the intrinsic NIR fluorescence signal of Ce6 released by the nanoprobes. About three weeks after MGC-803 cells injection, the size of tumor nodules was to 50–80 mm^3^. Free Ce6 (10 μg/mL, 100 μL) and MNPs-PEG_2K_-FA@Ce6 nanoprobes (equivalent Ce6 concentration) were intravenously injected in nude mice for real-time detection of the distribution *in vivo* of the drug. Figure SI12 showed that strong fluorescence was mainly present in the liver using free Ce6-treated mice. However, the signal in the tumor tissue was relatively weak because of the absence of tumor specific targeting. Additionally, the fluorescence signal in mice decreased rapidly at 24 h post-injection attributed to the small size and poor pharmacokinetics of Ce6 micromolecule. Nevertheless, we also observed a distribution of strong fluorescent signal in the liver and lungs at 12 h post-injection, higher tumor targeting was displayed for the MNPs-PEG_2K_-FA@Ce6 nanoprobes injected into the mice. The enhanced fluorescent intensity in tumor was sustainable for 24 h, fully proving that MNPs-PEG_2K_-FA@Ce6 nanoprobes were not rapidly eliminated from the body. With increasing time, the fluorescent intensity of the tumor areas became more distinct at 1 d post-injection due to the nanoprobes accumulation in the tumors, but meanwhile the fluorescence background of other organs had weakened. Interestingly, a fluorescent signal was almost undetected in liver tissue after two days. Figure 7a showed that the fluorescent signal in the tumor lasted for more than 6 d, indicating that MNPs-PEG_2K_-FA@Ce6 nanoprobes had the relatively very long tumor retention time attributed to active targeting and EPR effect. We speculate that an acidic environment in tumor can promote the corrosion of the Fe_3_O_4_ cores of nanoprobes, leading to the release of drug molecules^32^,^33^,^34^. So it confirmed that there was gradually specific release of Ce6 in tumor. The corrosion behaviour could result in removal and dissociation of the partial PEG_2K_ coating. The degradation of the organic coating could be similar to proteolytic digestion^33^. In particular, high tumor targeting efficiency of MNPs-PEG_2K_-FA@Ce6 nanoprobes was also vividly indicated via fluorescence images of isolated viscus at 24 h post-injection (Fig. 7b,c). As shown in Fig. 7c, although certain MNPs-PEG_2K_-FA@Ce6 nanoprobes appeared in normal tissues including heart, liver, spleen and so on at 24 h post-injection, there would be almost no toxicity of MNPs-PEG_2K_-FA@Ce6 nanoprobes for normal organs without NIR laser irradiation.

**Figure 8:**
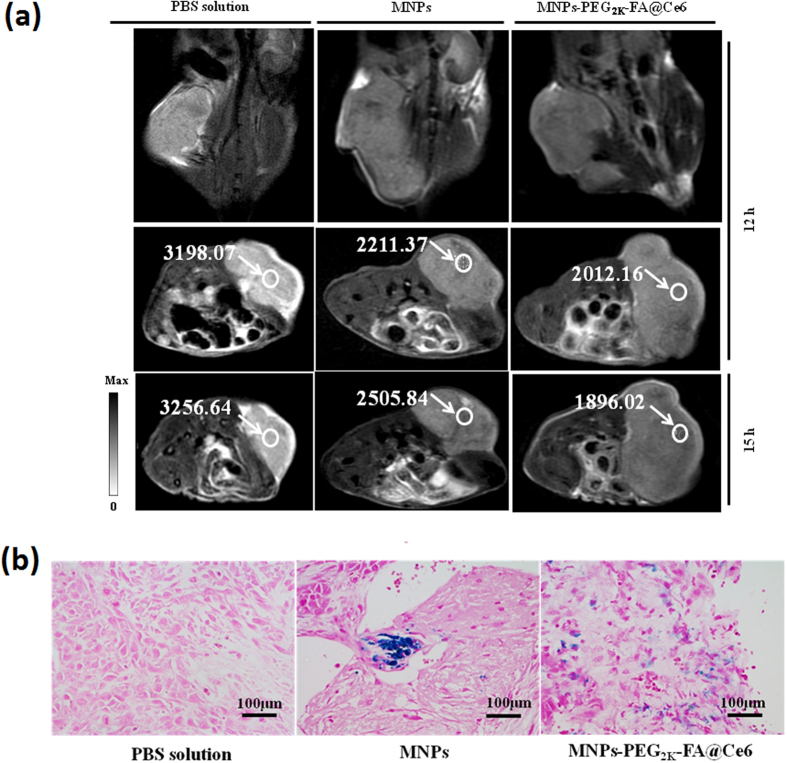
*In vivo* MRI. (**a**) MR images acquired at 12 h and 15 h - post injection of MNPs and MNPs-PEG_2K_-FA@Ce6 under the T2-weighted. (Note that: White circles and arrows indicate tumors in the presence of MNPs and targeted nanoprobes, respectively. These numbers respect T2-weighted mean signal intensity). (**b**) Representative Prussian Blue staining images of tumor cryosections (10 μm) at 12 h post-injection. Images presented are blank, MNPs and MNPs-PEG_2K_-FA@ Ce6 nanoprobes.

To further investigate the use of MNPs-PEG_2K_-FA@Ce6 as an effective imaging reagent, MRI was carried out at 12 h and 15 h after intravenous injection of the nanoprobes (Fig. 8a). Notably, after 12 h and 15 h post-injection the MNPs or the MNPs-PEG_2K_-FA@Ce6, the MR signal in the tumor mass displayed darker than that of blank control group. With the injection of the no-targeted MNPs, the signal value gradually increases from 69.1% (12 h) to 76.9% (15 h). In comparison, the signal value of the tumor injected with the targeted Fe_3_O_4_-PEG_2K_-FA@Ce6 nanoprobes gradually decreases from 62.9% (12 h) to 58.2% (15 h). Most importantly, compared to no-targeted MNPs, the T2-MR images at 12 h post-injection of the targeted MNPs-PEG_2K_-FA@Ce6 nanoprobe appeared to be more homogenously distributed in the tumor region (Fig. 8b), which implies that the FA-modified MNPs-PEG_2K_-FA@Ce6 nanoprobes can particularly target the overexpression of membrane folate receptors according to a receptor-mediated manner and have relatively long tumor retention time, consistent with the fluorescence imaging results. This result indicates that PEG coatings and FA ligands were introduced to slow down protein adsorption and nanoparticle aggregation, thus increasing the nanoparticle biocompatibility and promoting their internalization acting on targeted tumor, thus contributed to efficient photodynamic therapy.

### Photodynamic therapeutic efficacy of MNPs-PEG_2K_-FA@Ce6 *in vivo*

**Figure 9:**
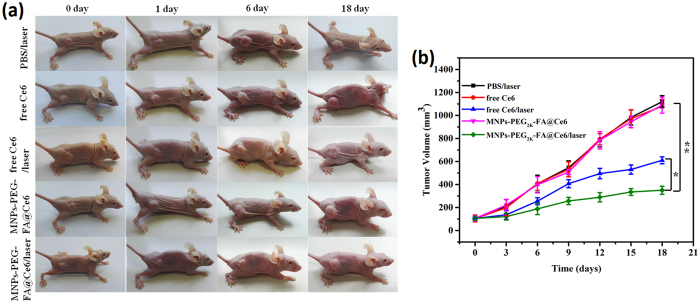
*In vivo* photodynamic therapy of MNPs-PEG_2K_-FA@Ce6. (**a**) The optical photograph of different groups before and after treatments. The solid tumor sites of tumor-bearing mice respectively treated using PBS, free Ce6 and MNPs-PEG_2K_-FA@Ce6 were irradiated with 633 nm laser (50 mW/cm^2^) for 30 min after 4 and 24 h post-injection. (**b**) MGC-803 tumor growth curves of different groups after treatments. The data are shown as mean ± SD (n = 5).

To evaluate the photodynamic therapeutic efficacy of MNPs-PEG_2K_-FA@Ce6 *in vivo*, the changes of tumors in volume were inspected over 18 days. After 4 and 24 h post-injection, the solid tumor sites of tumor-bearing mice respectively treated using PBS, free Ce6 and MNPs-PEG_2K_-FA@Ce6 were irradiated with 633 nm laser (50 mW/cm^2^) for 30 min, separately. As shown in Fig. 9, compared with normal group, the growth of MGC-803 tumors treated with MNPs-PEG_2K_-FA@Ce6/laser in mice was significantly inhibited by ~69%, illustrating the effectiveness of the photodynamic therapy. In control group, mice receiving free Ce6 plus laser treatment showed ~46% tumor growth inhibition. While the growth inhibition of tumor on mice treated with free Ce6 without laser and MNPs-PEG_2K_-FA@Ce6 without laser showed merely below 5%. It may be due to the long tumor residence time and selective tumor-targeting capabilities of MNPs-PEG_2K_-FA@Ce6 nanoprobes.

**Figure 10:**
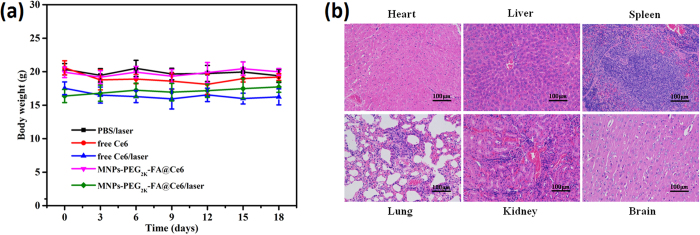
No noteworthy sign of toxic side effect of MNPs-PEG_2K_-FA@Ce6/laser *in vivo*. (**a**) Time-independent body weight curves of mice after various treatments. The data are shown as mean ± SD (n = 5). (**b**) H&E stained tissue sections of major organs including liver, spleen, lungs, kidneys and heart of the mice treated with MNPs-PEG_2K_-FA@Ce6/laser after day 18. Scale bar, 100 μm.

To confirm and investigate the potential toxicity of MNPs-PEG_2K_-FA@Ce6 in mice, the changes of tumor-bearing mice in weight were monitored as an indicator of poisonous side effect measurement. As shown in Fig. 10a, no noticeable change in mice weight was observed in five groups. At day 18, the major organs and tissues (liver, spleen, lungs, kidneys, heart and brain) collected after injection in MNPs-PEG_2K_-FA@Ce6/laser treatment group were sliced, stained with hematoxylin and eosin (H&E) with the standard protocol, then it was observed with microscopy. Notably, no pathological abnormalities or other conditions were observed by histological examination of major organs tissues in our experiments (Fig. 10b). Clearly, no noteworthy sign of toxic side effect of MNPs-PEG_2K_-FA@Ce6/laser *in vivo* was observed, as further uncovered by histological examination after receiving photodynamic therapy. Considering the less accumulation of MNPs-PEG_2K_-FA@Ce6 in the liver after more than 6 days, it may be logical to conclude that nanoprobes would not lead to significant long-cycle side effect in the body.

## Conclusions

In conclusion, we have designed a convenient and feasible method for magneto/fluorescent dual-modal imaging of gastric cancer by introducing MNPs-PEG_2K_-FA@Ce6 synthesized in aqueous phase as a contrast agent with co-imaging effect. The fluorescence imaging and MR images vividly illustrate the distribution of nanoparticles in whole body, especially to determine the uptake quantity in normal tissue and tumor. It is possible not only to understand the tumor targeting mechanism of the contrast agent, but also to achieve a comprehensive medical diagnosis for the optimized treatment efficacy. Based on these results, the selective targeting of the tumor and long residence time of the nanoprobes were dramatically enhanced via MNPs modified by FA ligands and PEG_2K_. More importantly, penetration and retention time of more than 6 days with less accumulation in the liver due to the highly stable MNPs-PEG_2K_-FA@Ce6 nanoprobes was observed, avoiding damage to healthy organs or tissues due to drug toxicity. Moreover, under 633 nm laser irradiation, the significant therapeutic effect of these nanoprobes were exhibited *in vivo*. Based on these findings, the contrast agent with co-localization effect could be potentially used for tumor recognition and monitoring before and after treatment in clinical medicine, may prospectively serve as a novel photodynamic anticancer nanoprobe in the near future.

## Additional Information

**How to cite this article**: Yin, T. *et al*. Superparamagnetic Fe_3_O_4_-PEG_2K_-FA@Ce6 Nanoprobes for *in Vivo* Dual-mode Imaging and Targeted Photodynamic Therapy. *Sci. Rep*. **6**, 36187; doi: 10.1038/srep36187 (2016).

**Publisher’s note:** Springer Nature remains neutral with regard to jurisdictional claims in published maps and institutional affiliations.

## Supplementary Material

Supplementary Information

## Figures and Tables

**Figure 1:**
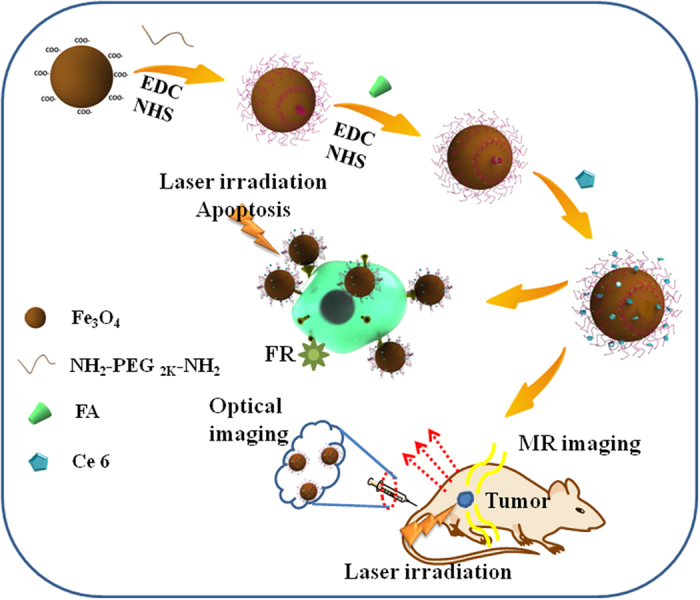
Schematic of MNPs-PEG_2K_-FA@Ce6 nanoprobes for *in vitro/vivo* imaging.
