# Supplementary material for: Superparamagnetic Fe3O4-PEG2K-FA@Ce6 Nanoprobes for in Vivo Dual-mode Imaging and Targeted Photodynamic Therapy
Source: Sci Rep. 2016 Nov 8;6:36187. doi: 10.1038/srep36187 (PMC5099938; doi:10.1038/srep36187)
Supplement: Supplementary Information [file srep36187-s1.doc]

Superparamagnetic Fe_3_O_4_-PEG_2K_-FA@Ce6 Nanoprobes for in Vivo Dual-mode Imaging and Targeted Photodynamic Therapy

Ting Yin^a^, Peng Huang^a^, Guo Gao^a^,*, Joseph G. Shapter^b^, Yulan Shen^c^, Rongjin Sun^a^, Caixia Yue^a^, Chunlei Zhang^a^, Yanlei Liu^a^, Sui Zhou^a^ and Daxiang Cui^a^,*

AUTHOR ADDRESS: ^a^Institute of Nano Biomedicine and Engineering, Shanghai Engineering Research Center for Intelligent Diagnosis and Treatment Instrument, Department of Instrument Science and Engineering, Department of Micro/Nano Electronics, School of Electronic Information and Electrical Engineering, Shanghai Jiao Tong University, 800 Dongchuan Road, Shanghai 200240, P. R. China.

^b^School of Chemical and Physical Sciences, Flinders University, Bedford Park, Adelaide 5042, Australia.

^c^Department of Radiology, Shanghai Jiao Tong University Affiliated Sixth People’s Hospital, Shanghai Jiao Tong University, Shanghai, 200240, China.

* To whom correspondence should be addressed. Tel: 0086-21-34206886; Fax: 0086-21-34206886; Email: [guogao@sjtu.edu.cn](mailto:guogao@sjtu.edu.cn) (G. Gao), [dxcui@sjtu.edu.cn](mailto:dxcui@sjtu.edu.cn) (D. Cui)

**
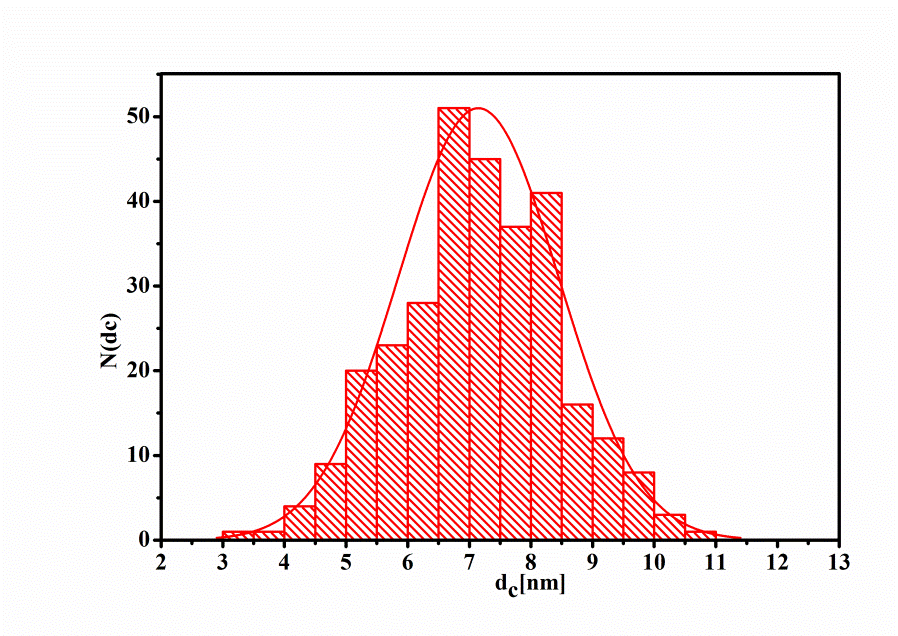
**

**Figure S1.** The diameter distribution of MNPs

**Conductometric titration**

About 1 mL HCl (0.1 mol/L) solution was added into 50 mL MNPs nanoparticle suspension (solid content is about 150 mg) and stirred at 25 °C for 10 min. Then a 0.01 mol/L NaOH aqueous solution was used as titrant. The amount of surface carboxyl groups is calculated from the break in curves and expressed in terms of milliequivalent of carboxyl groups per 1 g of the microspheres [30](#_ENREF_30):

—COO^-^ (mmol/g) = C · V / W

Where C and V are the concentration and volume of titrant, respectively, and W is the solid content weight of the Fe_3_O_4_ nanoparticles. The amount of carboxyl groups on the surface of the Fe_3_O_4_ nanoparticles are 0.73 mmol/g.


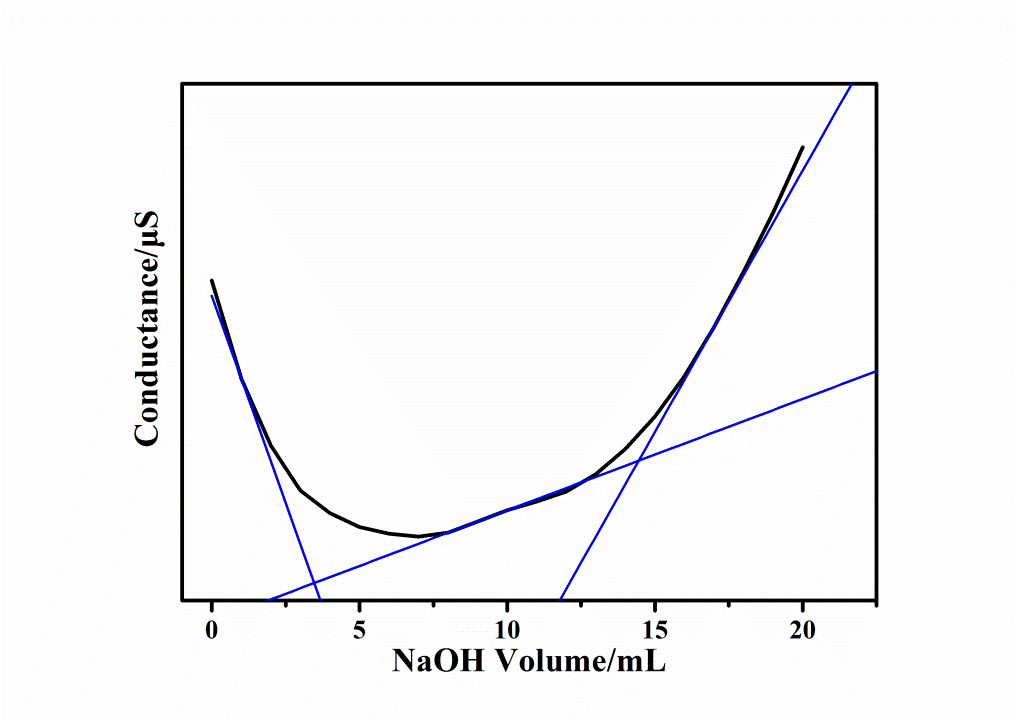


**Figure S2.** Conductometric titration curve of carboxyl groups


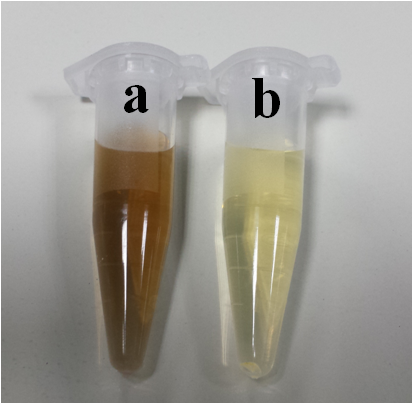


**Figure S3.** Visual image of (a) MNPs and (b) MNPs-PEG_2K_-FA@Ce6 nanoprobes dispersed in phosphate buffered saline


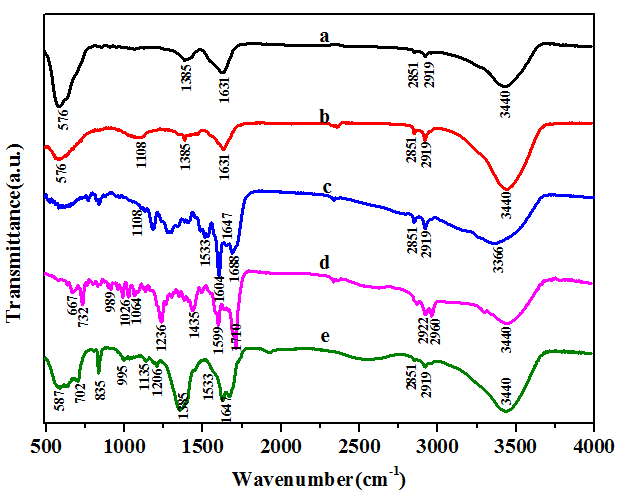


**Figure S4.** FTIR spectra of a) MNPs, b) MNPs-PEG_2K_, c) MNPs-PEG_2K_-FA, d) Ce6, and e) MNPs-PEG_2K_-FA@Ce6


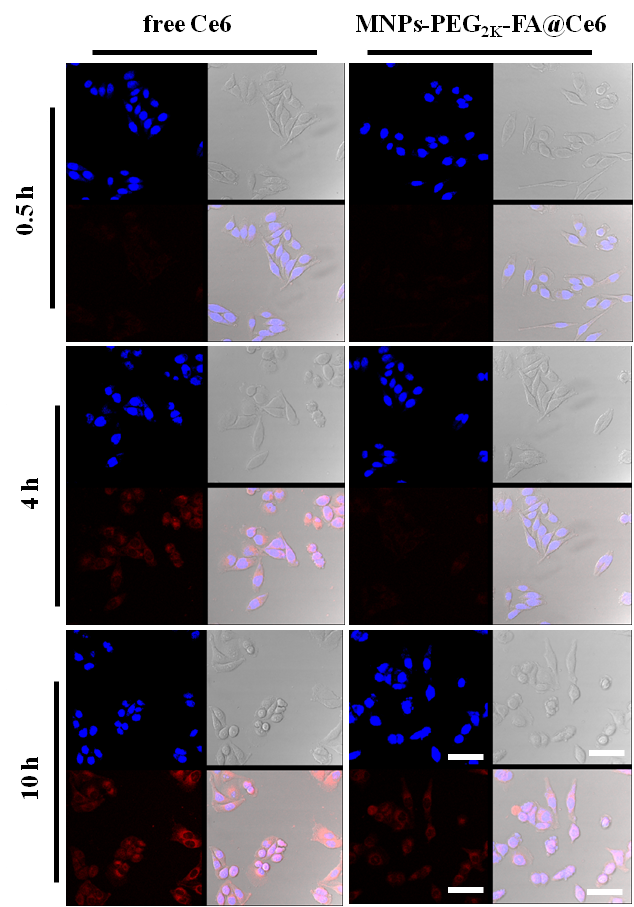


**Figure S5.** Confocal images of GES-1 cells exposed to free Ce6 or MNPs-PEG_2K_-FA@ Ce6 for 0.5h, 4 and 10 h. (Note that: Each block diagram was composed of four small squares. Top left of four small squares was fluorescent images of cells’ nucleus in blue field; lower left image was fluorescent images about cellular uptake of MNPs-PEG_2K_-FA@ Ce6 or free Ce6 in red field; top right images are of GES-1 cells in light field; lower right are merge images of the other three small squares). The scale bar was 100μm.


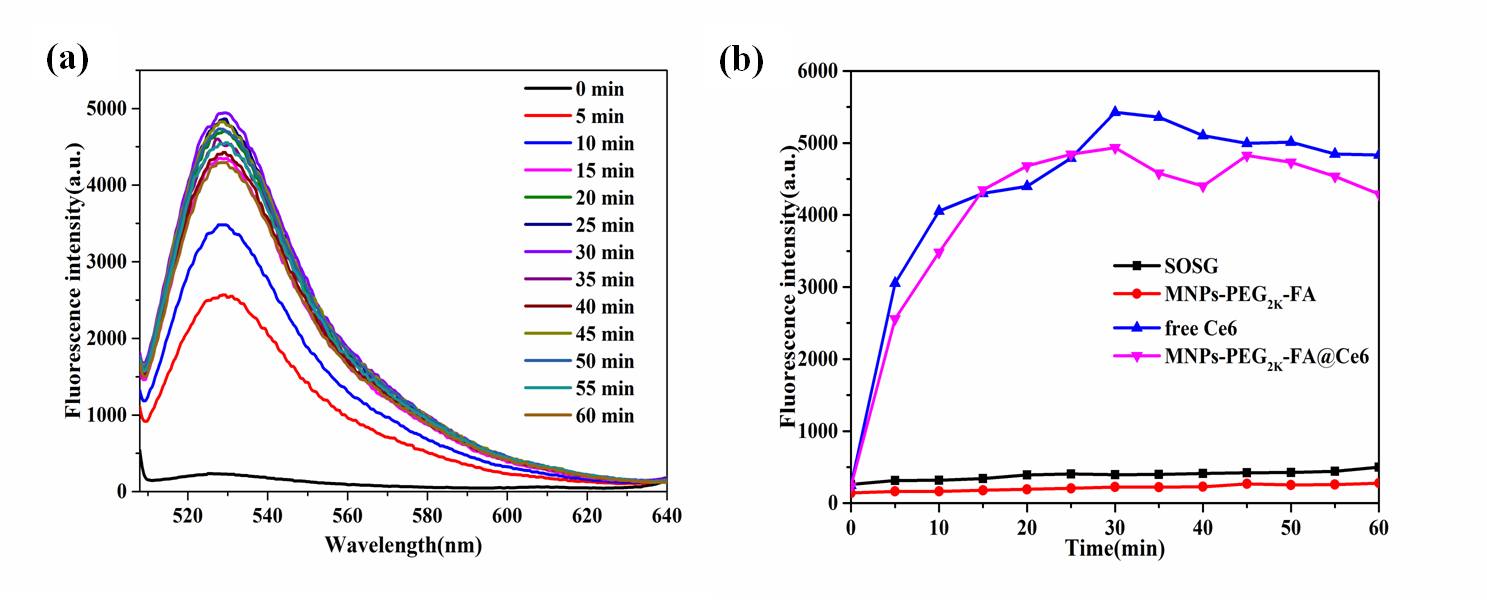


**Figure S6.** Singlet oxygen detection test using a singlet oxygen sensor green(SOSG) reagent.(a) Time-dependent fluorescence spectra (λ ex=494nm) of the SOSG/MNPs-PEG_2K_-FA@Ce6 mixture with light irradiation at 633nm (30mW/cm^2^). (b)The changes in fluorescent intensity at the characteristic peak of SOSG (528nm) as the function of light exposure time. Data were recorded every 5 min over 60 min period.


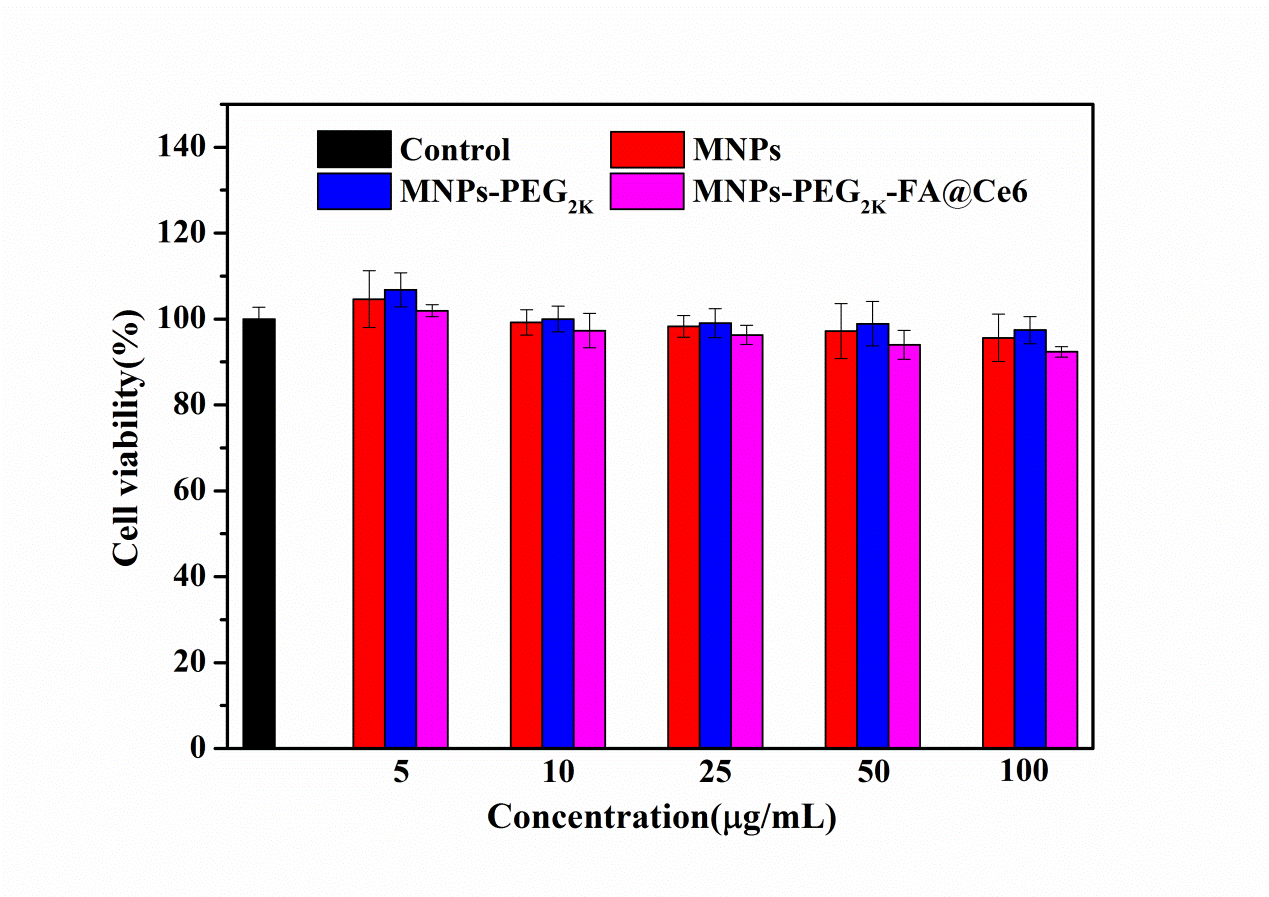


**Figure S7.** The toxicity of MNPs, MNPs-PEG_2K_ and MNPs-PEG_2K_-FA @ Ce6 toward MGC-803 cells, analyzed by MTT assay.


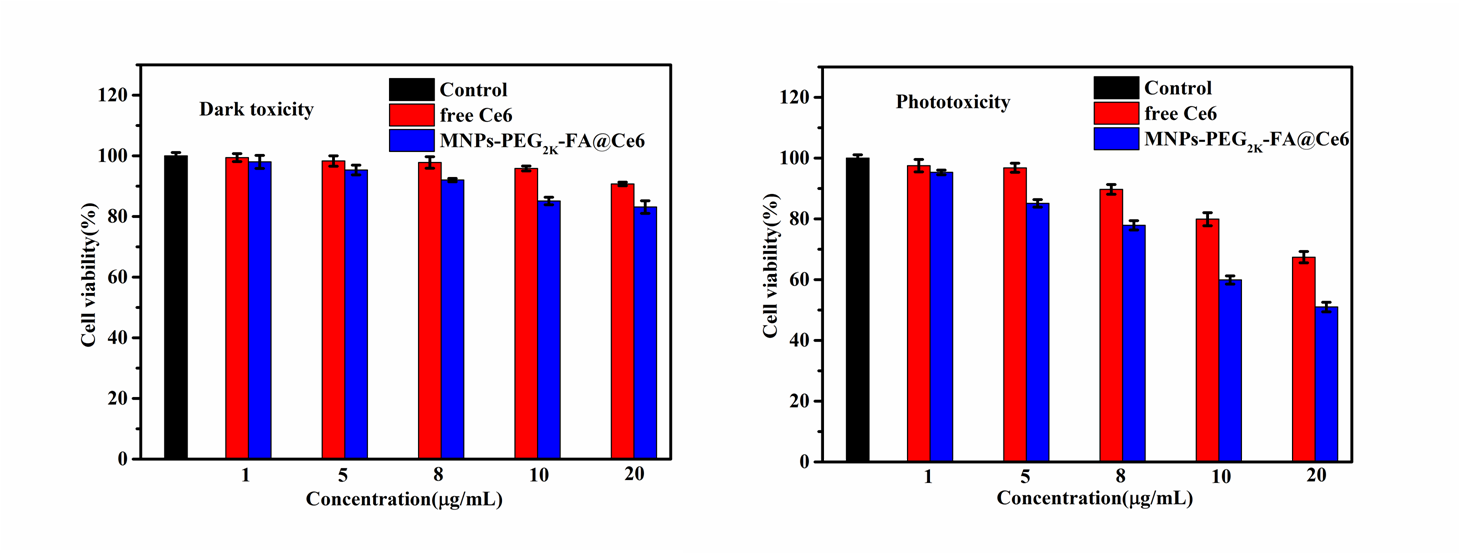


**Figure S8.** The toxicity analysis in the dark (left) and under the laser irradiation (λ_ex_=633nm) (right) of free Ce6 and MNPs-PEG_2K_-FA @ Ce6 incubation with GES-1 cells.


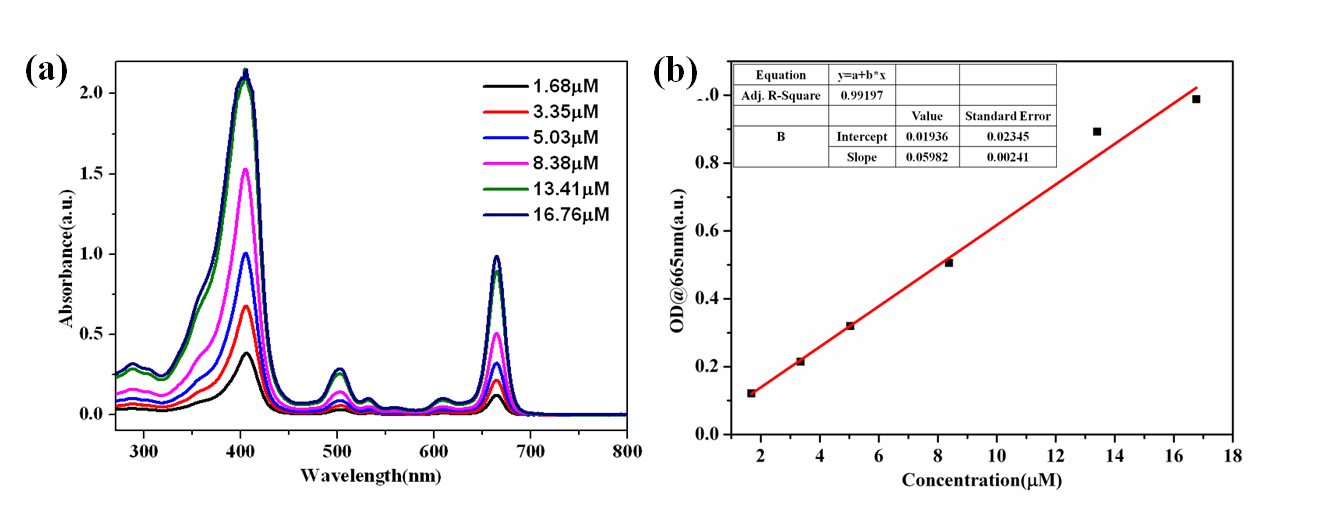


**Figure S9.** UV-vis absorbance spectra of (a) Ce6 at different concentrations, (b) Ce6 UV calibration curve at 665 nm.


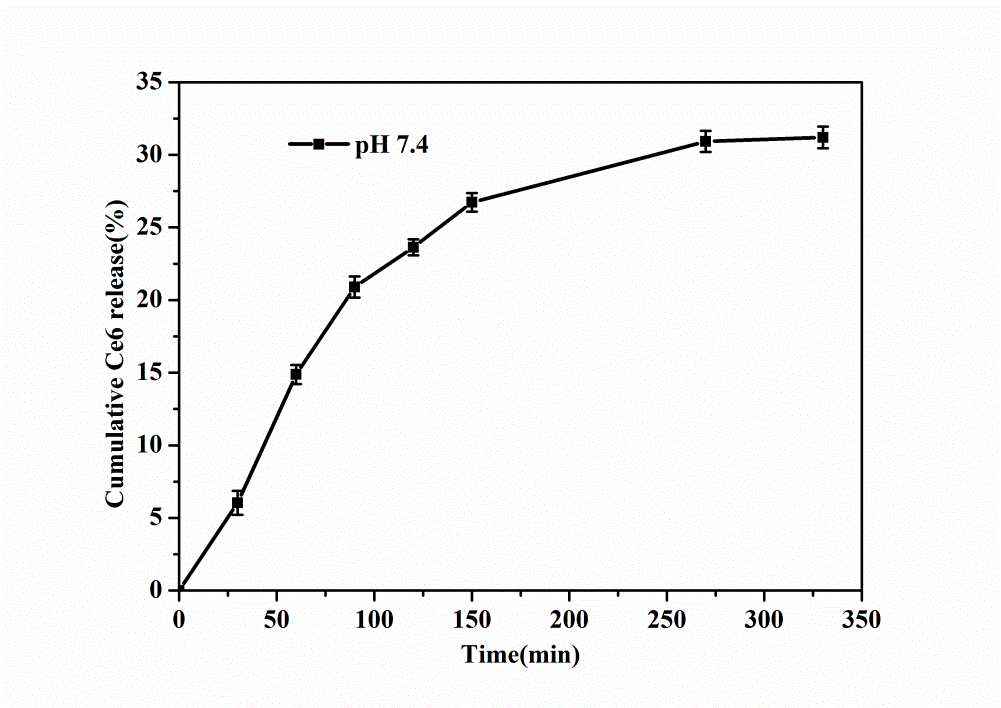


**Figure S10.** Release curve of Ce6 from MNPs–PEG_2K_-FA@Ce6 PBS with pH=7.4 values. Error bars were based on SD of triplicated samples.


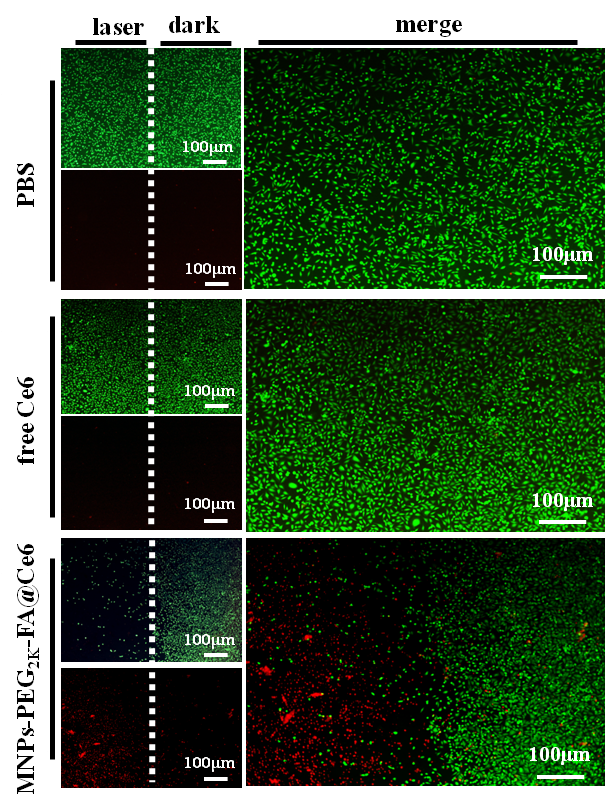


**Figure S11.** Phototoxicity of free Ce6 and MNPs-PEG_2K_-FA@ Ce6 against MGC-803 cells. Detection of photodamage by fluorescence microscopy using fluorescent probes (double-staining with calcein PI andcalcein-M). Dead cells: red fluorescence of PI; live cells: green fluorescence of calcein-AM. The left side of the dotted line was irradiated while the right side was in the dark.


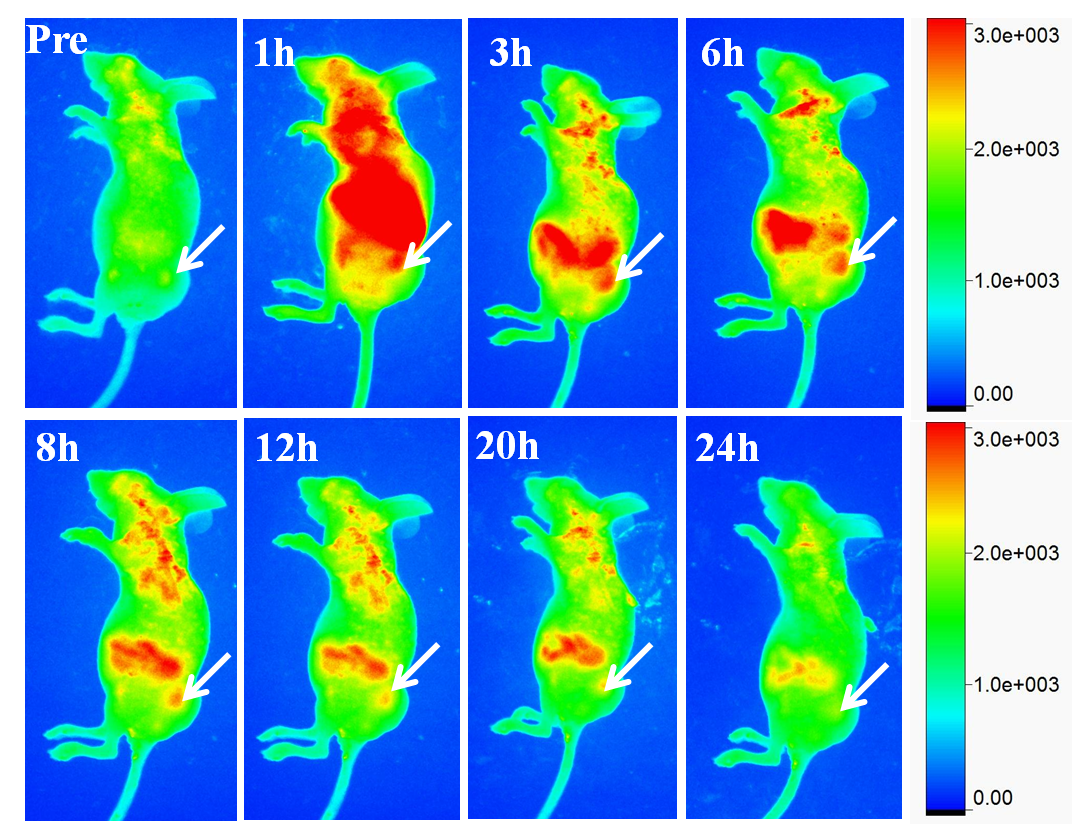


**Figure S12.** In vivo fluorescence imaging of MGC-803 tumor-bearing mouse after intravenous injection of free Ce6 over a period of 24 h. Though free Ce6 had some passive tumor targeting accumulation, the poor pharmacokinetics made them cleared from the body less than 24 h. These results clearly demonstrated small dye molecules are easily metabolized by the mice.
